# Micromotor-based dual aptassay for early cost-effective diagnosis of neonatal sepsis

**DOI:** 10.1007/s00604-023-06134-x

**Published:** 2024-01-19

**Authors:** José M. Gordón Pidal, Luis Arruza, María Moreno-Guzmán, Miguel Ángel López, Alberto Escarpa

**Affiliations:** 1 Department of Analytical Chemistry, Physical Chemistry and Chemical Engineering, Faculty of Sciences, University of Alcalá, Ctra. Madrid-Barcelona, Km. 33.600, Alcalá de Henares, 28802 Madrid, Spain; 2 Department of Neonatology, Instituto del Niño y del Adolescente, Hospital Clínico San Carlos-IdISSC, 28040 Madrid, Spain; 3 Department of Chemistry in Pharmaceutical Sciences, Analytical Chemistry, Faculty of Pharmacy, Complutense University of Madrid, Plaza Ramón y Cajal, S/N, 28040 Madrid, Spain; 4 Chemical Research Institute “Andrés M. Del Rio”, University of Alcalá, Madrid, Spain

**Keywords:** Procalcitonin, Interleukin-6, Sepsis biomarkers, Aptamers, Fluorescence microscopy, Bedside/point-of-care technologies

## Abstract

Given the long-life expectancy of the newborn, research aimed at improving sepsis diagnosis and management in this population has been recognized as cost-effective, which at early stages continues to be a tremendous challenge. Despite there is not an ideal-specific biomarker, the simultaneous detection of biomarkers with different behavior during an infection such as procalcitonin (PCT) as high specificity biomarker with one of the earliest biomarkers in sepsis as interleukin-6 (IL-6) increases diagnostic performance. This is not only due to their high positive predictive value but also, since it can also help the clinician to rule out infection and thus avoid the use of antibiotics, due to their high negative predictive value. To this end, we explore a cutting-edge micromotor (MM)-based OFF–ON dual aptassay for simultaneous determination of both biomarkers in 15 min using just 2 μL of sample from low-birth-weight neonates with gestational age less than 32 weeks and birthweight below 1000 g with clinical suspicion of late-onset sepsis. The approach reached the high sensitivities demanded in the clinical scenario (LOD_PCT_ = 0.003 ng/mL, LOD_IL6_ = 0.15 pg/mL) with excellent correlation performance (*r* > 0.9990, *p* < 0.05) of the MM-based approach with the Hospital method for both biomarkers during the analysis of diagnosed samples and reliability (Er < 6% for PCT, and Er < 4% for IL-6). The proposed approach also encompasses distinctive technical attributes in a clinical scenario since its minimal sample volume requirements and expeditious results compatible with few easy-to-obtain drops of heel stick blood samples from newborns admitted to the neonatal intensive care unit. This would enable the monitoring of both sepsis biomarkers within the initial hours after the manifestation of symptoms in high-risk neonates as a valuable tool in facilitating prompt and well-informed decisions about the initiation of antibiotic therapy.

These results revealed the asset behind micromotor technology for multiplexing analysis in diagnosing neonatal sepsis, opening new avenues in low sample volume-based diagnostics.

**Graphical Abstract:**

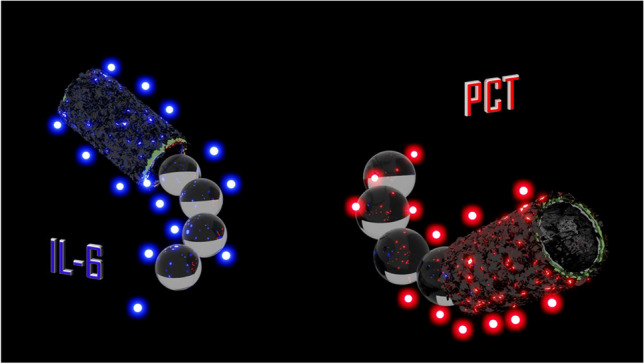

**Supplementary Information:**

The online version contains supplementary material available at 10.1007/s00604-023-06134-x.

## Introduction

Sepsis is a systemic inflammatory response caused by the presence of microorganisms in the bloodstream, causing more than 11 million deaths per annum worldwide [1]. Although anyone can suffer from sepsis, neonates increase mortality and the risk of adverse neurodevelopmental outcomes with all the associated social and economic burdens. Given the long-life expectancy of the newborn, research aimed at improving sepsis diagnosis and management in this population has been recognized as a cost-effective strategy for several reasons [2, 3]. As with older children and adults, the associated high mortality rates in neonates lead to early initiation of antibiotic treatment when sepsis is suspected and only a minority of infants are at last affected by sepsis. This implies a significant economic impact due to the high consumption of healthcare resources. In addition, this practice can potentially contribute to the appearance of resistance to antibiotics when they are incorrectly indicated. On the other hand, treatment delay in cases of true sepsis may be accompanied by lifelong sequelae in surviving preterm infants, such as neurodevelopmental delay, with all the associated economic and social costs. Therefore, an early diagnosis of sepsis is essential since late treatment increases the possibility of death. The definitive diagnosis of sepsis is established by the growth of bacteria in blood cultures, which is considered the gold standard diagnostic test for this disease. However, one of the main drawbacks in the diagnosis of sepsis in newborns by blood culture is the need to use relatively high volumes concerning their weight, the long analysis times (from several hours to days), as well as the variability inherent in this type of culture, especially when a limited clinical sample is used [4–6]. Furthermore, the obtention of the blood sample by venipuncture may be challenging in extremely preterm infants due to the tiny veins of these babies.

Specific blood biomarkers detection constitutes an interesting tool in addition to blood cultures since they can be found at high levels in the organism at the onset of the disease, even before the development of clear symptoms. The use of certain biomarkers such as PCT or IL-6 can help in a rapid diagnosis of true cases of sepsis, due to their high positive predictive value, but it can also help the clinician to rule out infection and thus avoid the use of antibiotics, due to their high negative predictive value. As we propose in our study, the multiplexed determination of biomarkers has been shown to increase diagnostic accuracy [7, 8]. This can be explained by the different behavior of each biomarker during infection [9]. For example, PCT is probably recognized as the most specific sepsis biomarker. Its levels begin to rise around 4 h after the onset of systemic infection and peak between 8 and 24 h. Although PCT may rise in certain non-infectious conditions, persistently normal values, and a lack of rise in the hours after symptom onset have a very high negative predictive value. Therefore, PCT is a very interesting biomarker to rule out infections and avoid unnecessary antibiotics. In contrast, IL-6 is one of the earliest biomarkers in sepsis, increasing very soon after infection and peaking at around 2 h. This profile is very useful when blood samples are obtained very early in the course of the infection when PCT values are still negative. Repeated measurements in the first hours after symptom onset can further increase biomarkers' negative and positive predictive values [10]. However, in the neonatal population, this is at the cost of the low blood volume of extremely preterm infants, making it impractical in real life in most cases. This blood volume limitation increases with the combined analysis of different sepsis biomarkers due to the volume consumption required for each determination. Additionally, the turnaround time for PCT results is up to 3 h in some studies [10]. Furthermore, the obtention of blood samples via venipuncture for sepsis assessment in extremely preterm infants can be challenging, even in experienced hands, due to the small caliber of veins and poor peripheral perfusion of these critically ill neonates. The alternative then is to start and maintain antibiotics for several days before confirming the presence or absence of infection.

The literature has reported different approaches for individual determination of PCT and IL-6 such as chemiluminescent (PCT [11], IL-6 [12]), colorimetric (PCT [13], IL-6 [14]), electrochemical (PCT [15], IL-6 [16]), and fluorescence (PCT [17], IL-6 [18]) immunoassays. IL-6 was also detected by aptassays through AuNPs [19, 20], carbon nanotubes [21], and graphene nanosensors [22, 23]. But these assays are high-time and sample-consuming [20–23], and in some cases, sensitivity is not sufficient to meet the cut-off requirements [19, 24].

However, there are few studies in the bibliography on the dual detection of PCT and IL-6. Berger et al. reported the diagnosis through a microfluidic immunoassay with LODs of 130 pg/mL for PCT and 150 pg/mL for IL-6. In the case of IL-6, this LOD is quite far from the cut-off value (20 pg/mL). Besides, the approach was not able to quantify the levels of these biomarkers in plasma, only distinguishing between healthy and high-expression plasma samples [25]. Both proteins were also detected through surface-enhanced Raman spectroscopy (SERS) with LODs of PCT = 0.042 ng/mL and IL-6 = 0.54 pg/mL [26]. However, this assay needs a large amount of sample (200 µL), and the measure of both proteins was made separately. On the other hand, Wu et al. developed a microfluidics immunoassay combined with streptavidin–biotin-peroxidase nano complex-signal amplification system to detect PCT and IL-6 with low limits of detection of PCT (1.0 pg/mL). However, in the case of IL-6 (48.9 pg/mL) it did not reach the cut-off value and the full assay needs a long times (> 3 h) [27].

On the other hand, micromotor (MM) technology has added a new dimension to analytical biosensing [28]. The junction of their autonomous motion, and enhancement of fluid mixing with their functionalization capabilities, allow reliable analyte detection in microliter samples with improved analytical performance. Indeed, the MM biosensing approach relies on the continuous movement of receptor-on board on MM through complex samples in connection to a plethora of ‘on-the-move’ biomolecular recognition [29, 30]. Specifically, bubble-propelled catalytic micromotors [31, 32] can also enhance analyte–receptor interactions through the increased fluid transport and mixing associated with the MM swimming and their generated gas microbubbles tail (i.e., molecular oxygen after hydrogen peroxide dismutation on Pt-based catalyst). It is a clear advantage in comparison with common biosensors that rely on placing the sample droplet over the receptor-modified transducer and measuring the extent of the analyte–receptor interaction after incubation under static conditions, being diffusion-controlled assays, which is a key disadvantage in terms of analyte transport limitations under microscale [33]. Since enhancing mass transport by using convection at these scales is often challenging for increasing the performance of such assays, the MM approach becomes an interesting alternative to solving this issue at the microscale analysis.

As stated above, they can also be smartly functionalized with a plethora of receptors, and antibodies the most commonly used [34–37]. In the last years, our group has developed on-the-fly individual immunoassays for procalcitonin [17] and C reactive protein [38, 39] biomarkers in neonatal sepsis using MM-based approaches, revealing the analytical potency of this technology in this challenging field.

More importantly, the potency of MM-based biosensing should be understood considering the collective behavior that MMs generate in dissolution, collectively acting as a (bio)sensing swarm, significantly increasing the possibilities of improving the (bio)sensing event in ultra-reduced volumes of the clinical sample.

To continue exploring the analytical potential of MM technology in the biosensing of sepsis protein biomarkers, it has been considered convenient to study the possible advantages offered by aptamers over antibodies such as more chemical stability, more reproducibility between batches, and tailored tagging with loss of performance, with high selectivity yet. Besides, the capacity to realize the performance in one step, avoiding washing steps (which is extremely important in clinical diagnosis) and addition of extra “reagents” as detection antibodies and enzymatic substrate for detection, reducing the time of analysis, a critical point in neonatal sepsis too.

To this end, and after the successful individual development of both individual MM-based aptassays for PCT [40] and IL-6 [41], here we report for the first time, an MM-based dual aptassay for the simultaneous determination of PCT and IL-6, as highly relevant early sepsis biomarkers for the sake of moving forward a multiplexed diagnosis of sepsis in special patients such as neonates. Today it is still a challenge to make an early diagnosis of sepsis in premature newborns where the available sample volume is very low, and the short analysis time is essential. On the other hand, the multiplexing capabilities of MM for biosensing remain unexplored in complex clinical scenarios, because the transfer of assays from individual to multiplexed formats requires fine design in the development of the aptassay and optimization of each variable to achieve results like those obtained in a similar single assay. Given this, a rapid and ultra-small volume-consuming determination of a combination of 2 of the most highly relevant and reliable sepsis biomarkers (combining a high specificity biomarker (PCT) with another of early diagnostic alarm/diagnostic readiness (IL-6)) using still disruptive technology such as MM is a considerable advance in the diagnosis of sepsis in preterm infants, which allows serial measurements in the first hours of the onset of symptoms to promptly initiate treatment in cases of true infection and avoid the use of antibiotics in the absence of sepsis. Ultimately, this can improve outcomes and reduce costs.

The enormous potential of MM technology would also translate into bedside/point-of-care devices to monitor the evolution of various sepsis biomarkers in a few easy-to-obtain drops of heel stick blood samples from newborns admitted to the neonatal intensive care unit.

## Experimental

### Reagents and aptamers

The specific aptamers for PCT (5′-GCG GAT GAA GAC TGG TGT GTG GGG GAG GGG TGA GTT TTA GTG TTT TTG TTG GTT GGC GGC CCT AAA TAC GAG CAA C-3′) and IL-6 (5´-GTT GCT CGT ATT TAG GGC CGA GGT ACG AGT GTC TTT GGG ATC TGC ATT CTC CTG CGT GAC ACC AGT CTT CAT CCG C-3′) were synthesized by Aptus Biotech by SELEX technology. A proposed structure is given in Figure S1.

For SELEX, a chemically synthesized and HPLC-purified DNA oligonucleotide library (IBA Lifesciences, Goettingen, Germany) consisting of a pool of 76 nt-long oligonucleotides was used. This population was incubated with either recombinant human IL-6 (Abyntek Biopharma) or recombinant Human Procalcitonin (R&D Systems, Cat. No. 9607-PN), both fused to Histidine at the N-terminal end, previously immobilized on Ni–NTA Agarose resin (QIAGEN). The populations obtained after three selection rounds for IL-6 or six selection rounds for PCT were analyzed by real-time PCR to analyze the enrichment in specific sequences against the targets. Finally, massive sequencing (NGS—Illumina) was performed and the sequences of the aptamers against IL-6 and PCT were identified by Aptasuite [37].

Aptamer against IL-6 (Il6R1) was finally synthesized with Alexa488 6-FAM (Apt_IL-6_, *λ*_em_ = 520 nm) as a fluorescent label at 5′ whereas aptamer against PCT (PC61F) was synthesized with Alexa405 as a fluorescent label at 5′ (Apt_PCT_, *λ*_em_ = 447 nm) to perform the dual aptassay procedure.

Dilution of PCT and IL-6 was prepared in phosphate-buffered saline (PBS) buffer solution pH 7.5 (0.1 M Na_2_HPO_4_ (99%), 2.7 mM KCl (99%)) from Scharlau; 0.1 M NaH_2_PO_4_, 138 mM NaCl (99%) from Panreac. The aptamers were prepared in phosphate-buffered-MgCl_2_ pH 7.5 (PB MgCl_2_) (0.1 M Na_2_HPO_4_, 0.1 M NaH_2_PO_4_, and 1 mM MgCl_2_ from Sigma-Aldrich).

Graphene oxide (GO) (4 mg/mL dispersion in H_2_O), H_2_SO_4_ and Na_2_SO_4_, Nickel (II) sulfamate tetrahydrate (H_4_N_2_NiO_6_S_2_), Nickel (II) chloride hexahydrate (Cl_2_Ni-6H_2_O), hexachloroplatinic (IV) acid (H_2_PtCl_6_), dichloromethane, isopropanol, and ethanol were purchased from Sigma-Aldrich. Boric acid (99.5%) was purchased from Fluka. MicroPolish Alumina (0.05 µm) was purchased from Buehler, and hydrogen peroxide (H_2_O_2_) (30%) was purchased from Fisher Chemical. Five micrometer diameter conical pore polycarbonate membranes (PC) were purchased from Whatman. Bovine serum albumin (BSA) was purchased from Sigma-Aldrich and prepared in PBS buffer 0.1 M pH 7.5.

All chemicals used were analytical-grade reagents, and deionized water was obtained from a Millipore Milli-Q purification system (18.2 MΩ cm at 25 °C).

### Samples

Candidates for participation in the study were very preterm infants (< 32 weeks gestational age) of very low birth weight (< 1000 g) admitted to the neonatal intensive care unit (NICU) with clinical suspicion of late-onset sepsis (> 72 h of life). Symptoms suggestive of infection were the development of cardiorespiratory instability, apnea, thermal dysregulation, neurological deterioration, or ill appearance, in previously stable patients. Parents of babies meeting these criteria were approached to obtain informed consent to participate in the study. According to clinical practice, blood samples were obtained as part of sepsis evaluation for the determination of acute phase reactants levels (C-reactive protein, procalcitonin, and IL-6), white cell count, and blood cultures. Only if informed consent was obtained was an additional blood sample volume collected for investigation. Consequently, patient management was unaffected by the study and no procedures related to the investigation were done without parental permission. The study was approved by the local Ethics Committee (Ethics Committee for Clinical Research, Hospital Clinico San Carlos-IdISSC). Study approval reference code: 16/161-E, and code CEI: CEID2021/4/108 from the University of Alcalá.

Five hundred microliters of whole blood were collected in tubes with separator gel and transported to the laboratory of the Hospital for standard analysis where IL-6 was measured by chemiluminescent immunoassay using Cobas® e411 analyzer (Roche Diagnostics GmbH. D-68298. Mannheim Germany) and PCT by immunofluorescence with BRAHMS PCT sensitive KRYPTOR (B·R·A·H·M·S GmbH. Hennigsdorf, Germany). Another aliquot of 500 μL of whole blood was collected in tubes with a clot activator and gel for serum separation and centrifuged at 1000 rpm for 15 min. The supernatant was separated and transported to the research laboratory on ice.

### Apparatus

The MM electrosynthesis was performed using µ-Autolab Type III (Eco Chemie, Utrecht, Holland). Advanced VortexMixer-ZX3 from VWR and Thermosaker TS-100C from Biosan, and the Magnetic block DynaMag-2 from ThermoFisher were used for immunoassay incubation and magnetic MM handling, respectively. Scanning electron microscopy (SEM) images were obtained with a JEOL JSM 6335F and X-ray analysis was carried out through an EDX detector coupled to an SEM instrument. An inverted optical microscope (Nikon Eclipse 80i upright microscope), coupled with a Hamamatsu digital camera C11440, an objective (Nikon S Fluor 20X/0.75 DIC M/N2, ∞/0.17, WD 1.0), a DAPI 5060C filter for PCT detection (*λ*_ex_, 377 nm; *λ*_em_, 447 nm), and a B2-A filter for IL-6 detection (*λ*_ex_, 470 nm; *λ*_em_, 520 nm), was used for fluorescence measurements. The NIS Elements AR 3.2 software was used for capturing images and videos.

### Electrosynthesis of micromotors

Tubular graphene oxide (GO)/Ni/platinum nanoparticles (PtNPs) MM were synthesized by electrodeposition of three specific functional layers: inner-catalytic (PtNPs for bubble propulsion), intermediate-magnetic (Ni for magnetic handling), and outer-sensing (GO). S4-branched side of the membrane was previously treated with a sputtered thin gold film to perform it as a working electrode. Then, the membrane was assembled between a Teflon mold and a plating cell recovered with aluminum foil to make electrical contact with the working electrode. The electrodeposition of the three materials (GO, Ni, Pt) takes place through the following procedures: (i) The outer layer based on GO (0.5 mg/mL), previously dispersed in an ultrasonic bath for 15 min, was deposited through cyclic voltammetry (CV, + 0.3 to − 1.5 vs Ag/AgCl (3 M KCl), at 50 mV/s for ten cycles) using a Pt wire as the counter electrode. (ii) The intermediate layer of Ni was plated inside of the carbon layer by galvanostatic method (10 pulses of 20 mA for 0.1 s to generate nucleation spots, continued by a constant current of − 6 mA for 300 s to grow the Ni layer). (iii) The inner Pt layer that is plated inside the GO tube composed of 4 mM of H_2_PtCl_6_ and 0.5 M of acid boric was deposited by amperometry at − 0.4 V for 750 s. After the deposition of the three materials, the sputtered gold layer was hand polished with 0.3–1 µm alumina slurry to eliminate the material that does not form part of the MM. The cleaned membrane was washed with CH_2_Cl_2_ (3 washes for 15 min to completely release the MM); isopropanol (3 washes for 10 min); ethanol (2 washes) and ultrapure water (1 wash for 5 min). All MM were stored in ultrapure water at 4 °C when not in use. The template preparation method resulted in reproducible MM in shape, size, and motility.

### MM dual aptassay procedure

MM suspension was incubated with a highly specific aptamer against PCT (MM-Apt_PCT_ for 30 min) or IL-6 (MM-Apt_IL-6_ for 45 min) dissolved previously in PBMgCl_2_ (1 mM) to obtain a quenched MM-Apt_PCT_ or MM-Apt_IL-6,_ respectively. Subsequently, they were washed 3 times with PBS (50 µL) to remove the free aptamer. These washing steps are easily performed thanks to the magnetic Ni intermediate layer of MM that allows their retention by the aid of a magnet and proper handling into the microwell. Then, a final volume solution of 2 µL that contained a mixture of MM-Apt_PCT_ and MM-Apt_IL-6_ as well as PCT and IL-6 (1. 9 µL standard or sample) dissolved in BSA (5%) and the reagent for propulsion (H_2_O_2_, 2%; 0.1 µL of H_2_O_2_ 30%) was deposited in an ELISA microwell to perform the simultaneous *on-the-fly* recognition of both proteins by the autonomous movement of MM. After that, the solution was analyzed with an inverted optical microscope, using a DAPI 5060C fluorescence filter (*λ*_ex_, 377 nm; *λ*_em_, 447 nm) for PCT and a B2-A fluorescence filter (*λ*_ex_, 470 nm; *λ*_em_, 520 nm) for IL-6. The fluorescence signals obtained from both proteins were analyzed by the software associated with the microscope and fitted through the equation:

$$F=\left(\frac{{F}_{max}- {F}_{min}}{1+{\left(\frac{{EC}_{50}}{x}\right)}^{h}}+ {F}_{min}\right)$$

where *F* is the fluorescence signal, and coefficients *F*_max_ and *F*_min_ are the maximum and minimum fluorescence intensity values of the calibration graph, respectively. EC_50_ is the value of the analyte concentration corresponding to 50% of F_max_, and *h* is the hill slope.

LOD and LOQ were calculated as 3S/m and 10S/m, respectively; where *S* was the standard deviation (*n* = 10) obtained during the measurement of the fluorescence from the lowest PCT (0.01 ng mL^−1^) and IL-6 (0.5 pg mL^−1^) calibration concentrations, and *m* is the slope of the linear calibration plot.

## Results and discussion

### MM-based dual aptassay: the strategy

**Fig. 1:**
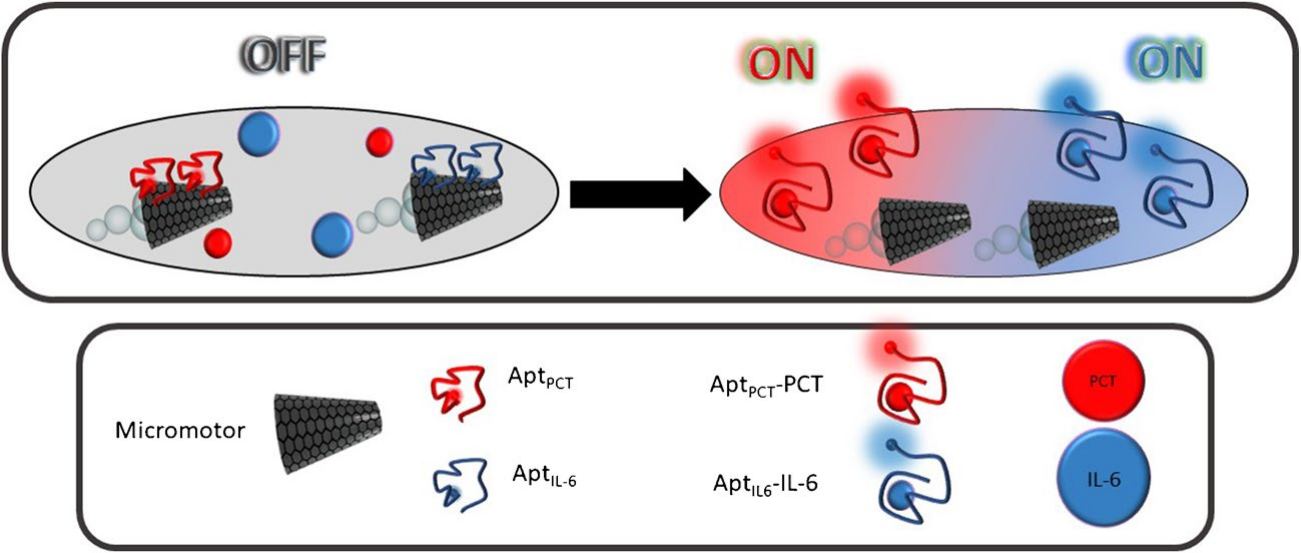
OFF–ON MM-based dual aptassay for simultaneous determination of PCT and IL-6

Figure 1 illustrates a scheme of the analytical strategy of the OFF–ON dual aptassay.

Specific PCT or IL-6 fluorophore-labeled aptamers are previously immobilized to MM through π-π bonds between ring structures from the nucleotide bases and the hexagonal cells of their GO outer layers (MM-Apt_PCT_) or IL-6 (MM-Apt_IL-6_). In these conditions, the GO surface properly quenches the fluorophores, corresponding to the “OFF” stage [42].

**Fig. 2:**
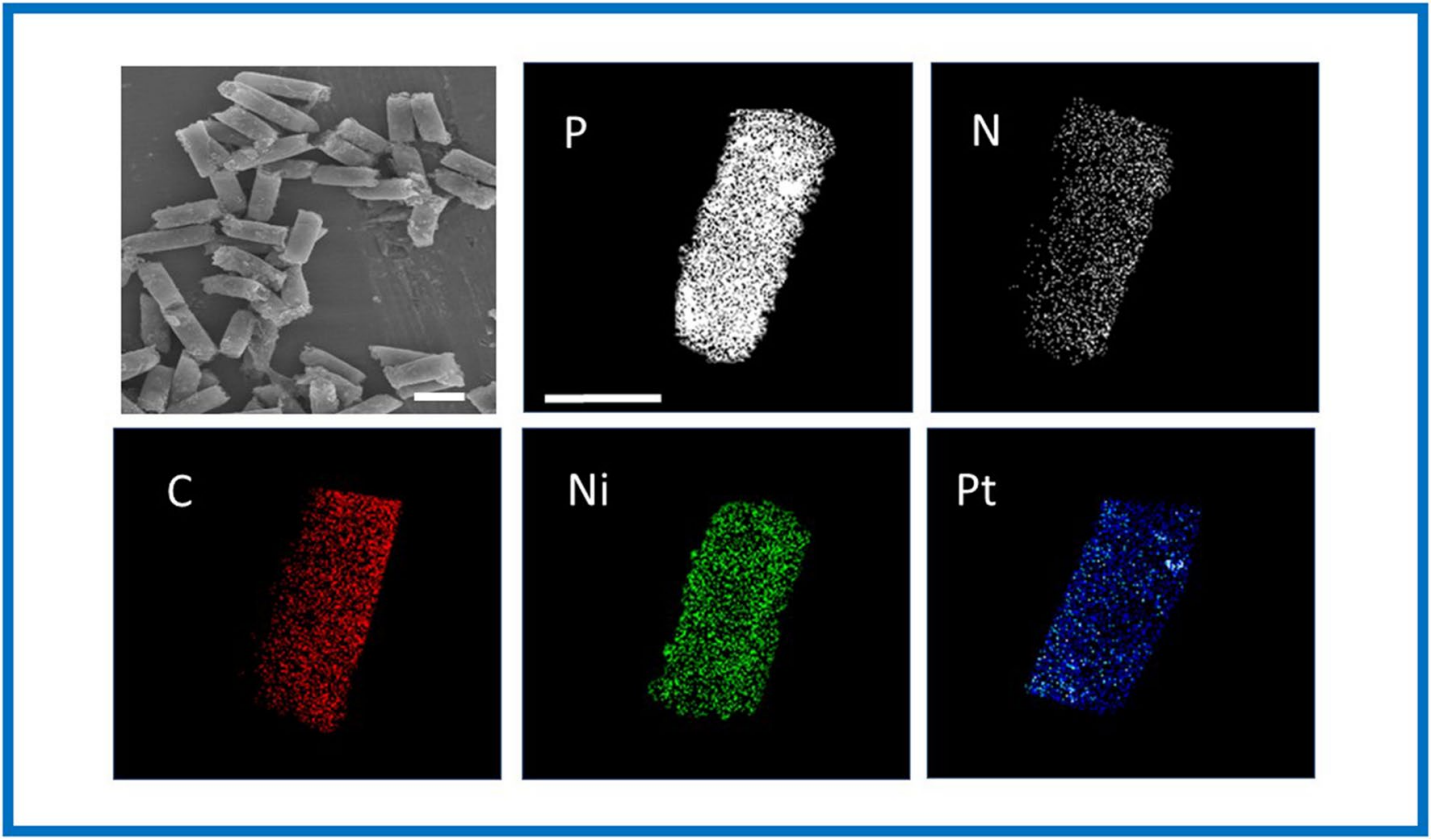
SEM images and EDX analysis of the GO/Ni/PtNPs MM. Scale bar: 10 µm SEM images and 5 µm EDX images

In the presence of each protein, their specific affinity interaction with the corresponding aptamer (PCT-Apt_PCT,447 nm_; IL-6-Apt_IL-6,520 nm_) causes a conformational change in the aptamer 3D structure, providing the desorption from the GO MM surface and, hence, recovering the fluorescence (“ON” stage). Figure 2 shows the characterization of GO/Ni/PtNPs MM using scanning electronic microscopy (SEM) images and X-ray spectroscopy analysis (EDX) to demonstrate the success of electrosynthesis and MM functionalization with the specific aptamers. GO/Ni/PtNPs MM displayed the structural morphology based on an almost conical shape with dimensions of 5 µm of width and 10 µm of length. Interestingly, the EDX analysis demonstrates the phosphorus and nitrogen content, confirming the presence of DNA on the surface of the MM from the phosphate groups and nitrogenous bases in the DNA backbone. In addition, the apparent loss of N and P after the interaction with the biomarker observed in the mapping was calculated based on the EDX spectra (not shown) with a remaining relative content of 20% P and 11% N (approx.). These amounts did not prevent obtaining reliable and reproducible quantitative results obtained in the analysis of clinical samples. More importantly, EDX mapping also confirmed the elemental composition of the MM homogeneously distributed (C as the biosensing layer, Ni as the magnetic layer, and Pt as the catalytic layer) demonstrating the efficiency of the MM electrosynthesis.

### MM-based dual aptassay: the optimization

Dual on-the-fly aptassay requires a fine optimization of variables involved in both OFF and ON stages since in this design, the detection of both PCT and IL-6 is performed simultaneously using the same ultra-reduced volume of the clinical sample. This implies challenges such as optimizing the adequate number of two independently decorated MM with their respective aptamers which influence the overall mixing phenomena and the properly aptamer desorption toward the formation of protein biomarker-Apt complex, choosing the adequate fluorophores, and more importantly demonstrating the capability to perform the simultaneous determination of both biomarkers with reliability, where they are at very low concentrations, with different order of magnitude (pg/mL for IL-6 and ng/mL for PCT), and all in one in the ultra-miniaturized clinical sample environment.

Table 1 lists the optimized values of the dual assay. While the variables that govern the OFF state are different for each biomarker (number of micromotors, aptamer concentration, and adsorption time of each aptamer onto the GO MM surface for quenching), those that govern the on-the-fly dual aptassay have been commonly optimized for the simultaneous determination of both biomarkers. Considering the diverse requirements for each assay, an analytical compromise was acquired not only in sensitivity but also in clinical sample volume and detection time (protein-Apt desorption and fluorescence recovery, ON state). Dual assays were evaluated by comparison of the signals obtained with and without the target protein. One of the challenges in the dual determination of IL-6 and PCT is that both biomarkers need to be determined with high sensitivity (0.25 ng/mL and 20 pg/mL cut-off values for PCT and IL-6 respectively in sepsis diagnosis) using low neonate sample volumes as possible. However, the sensitivity of IL-6 must be in the order of picograms while the PCT is in nanograms. This fact implies that the optimal sample volume (2 µL) found for the individual determination of IL-6 [41] has turned out to be the limiting one. The use of this low volume is crucial for sensitive IL-6 determination, while PCT analysis still presented enough sensitivity. For IL-6 analysis, higher sample volumes produced diminished fluorescence signals, a circumstance that is especially relevant for lower concentrations. This fact can be explained because of the dilution in the volume sample of the low amount of formed IL-6-Apt_IL-6,520 nm_ complexes when there are few molecules of the protein. Hence, both proteins can be determined using only 2 µL of clinical samples with enough sensitivity for their clinically relevant concentrations (Figure S2A).

**Table 1:**
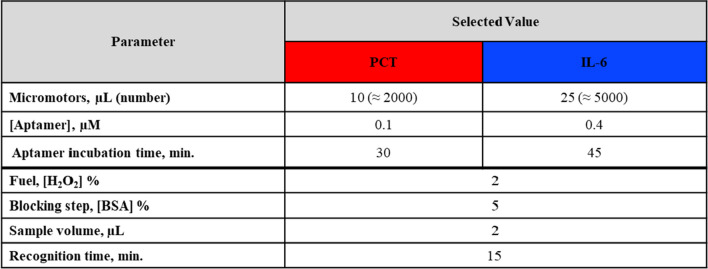
Optimization of GO/Ni/PtNPs MM-based dual aptassay for PCT and IL-6

Analysis times are another challenge in sepsis diagnosis since reliable results must be generated as soon as possible. The effective MM propulsion through the ejection of catalytically produced oxygen bubbles and the associated tail greatly improves the affinity interaction between the specific aptamers and sepsis biomarkers in very low sample volumes and short times. This fact can be confirmed in Figure S2B, where 15 min constituted the adequate interaction time to provide the highest signal for both proteins. Longer times implied diminished signals due to the increased oxygen concentration produced in the propulsion, which on the one hand gives rise to the formation of oxygen bubbles that make recognition and detection difficult, and due to its well-known behavior as a fluorescence quencher. In this case, the optimal analysis time (15 min) resulted in a time between 30 min necessary for the individual determination of IL-6 [41] and only 5 min necessary for the individual determination of PCT [40].

Figure S3 shows the incubation controls for PCT and IL-6 under bubble, static, and external stirring conditions. Quantitively, it is illustrated toward the signal ratio between the signals obtained in the presence (dark colors) and absence (light colors) of each protein biomarker. MM bubble propulsion improved biorecognition yields for both PCT and IL-6 compared to external stirring and static conditions since they produced the largest difference (approx. threefold) between the fluorescence signal (presence of protein) and the blank (absence of protein): 5.8 for PCT, and 5.2 for IL-6 vs. static (1.6 for PCT, and 1.9 for IL-6) and external stirring (1.9 for PCT, and 2.2 for IL-6). It also showed that the Apt-MMs approach decreased the unspecific adsorption. These results also show the effectiveness of the MM in generating an improved transport on the microscale that allows better mixing of fluids and therefore increases the efficiency of the immunoassay.

### MM-based dual aptassay: the analytical performance

**Fig. 3:**
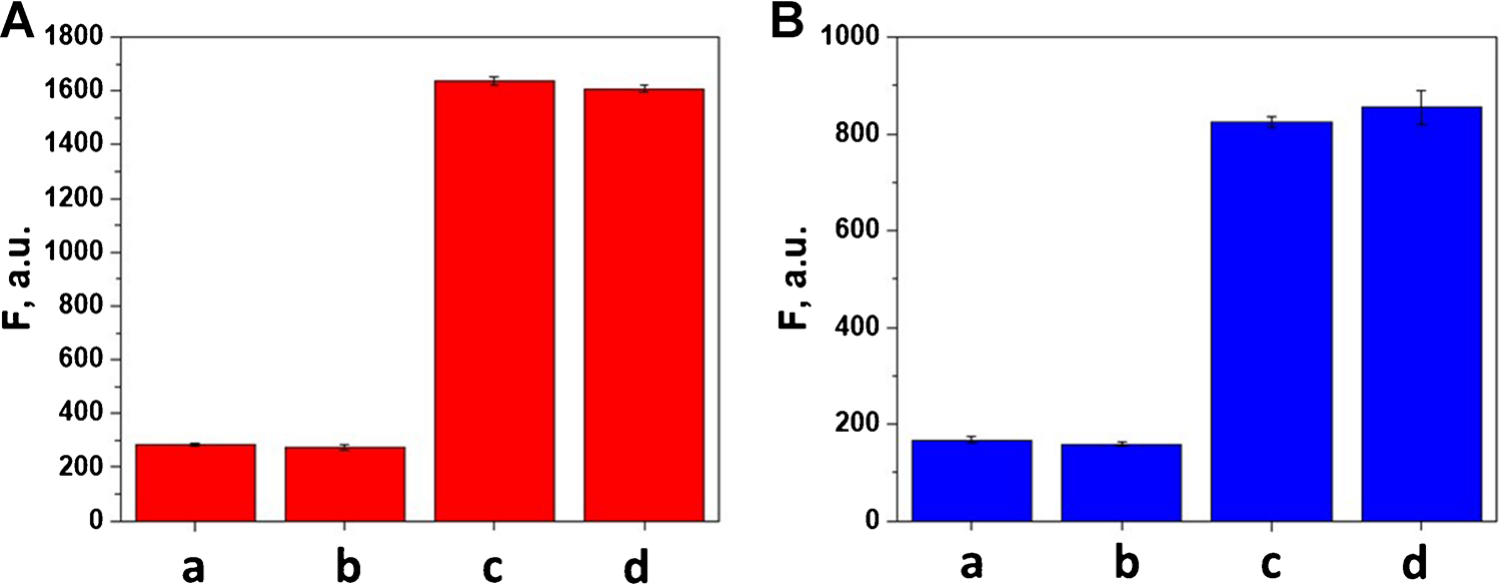
Cross-reactivity for dual aptassay of PCT (**A**) and IL-6 (**B**). For PCT: a) 0 PCT; b) 0 PCT + 2 µg/mL IL-6; c) 3 µg/mL PCT; d) 3 µg/mL PCT + 2 µg/mL IL-6. For IL-6: a) 0 IL-6; b) 0 IL-6 + 3 µg/mL PCT; c) 2 µg/mL IL-6; d) 2 µg/mL IL-6 + 3 µg/mL PCT. Fluorescence intensity was recorded at the selective wavelengths of aptamer-tagged labels (Alexa405 Apt PCT, *λ*_em_ = 447 nm and Alexa488 6-FAM) (Apt IL-6, *λ*_em=_520 nm), *n* = 3. Other conditions: (see Table 1)

Cross-reactivity is a crucial issue in the dual assay. As can be observed in Fig. 3, for each assay, there are barely any differences between the signals obtained in the absence and presence of the other protein, demonstrating excellent assay selectivity during the real-time dual assay where both MM are simultaneously navigating in just 2 μL of the same sample.

**Fig. 4:**
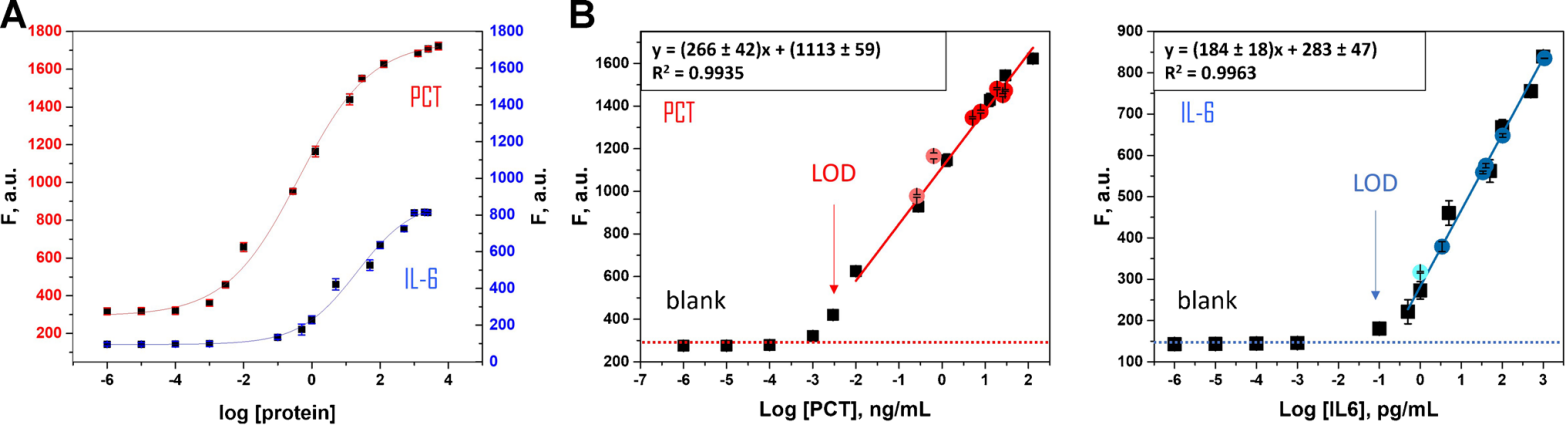
The sigmoidal curve of PCT and IL-6 (**A**) and the linear calibration plots (**B**) of PCT (left) and IL-6 (right) (in PBS pH 7.5). Clinical sample signal concentrations are depicted on each calibration plot (colored red for PCT and blue for IL-6 circles, *n* = 3). Conditions are described in Table 1

Table 2 lists the analytical features for the simultaneous determination of PCT and IL-6 and Fig. 4 shows the sigmoidal curves (A) and the calibration plots (in phosphate-buffered) (B) of PCT and IL-6. For PCT, the linear working ranged from 0.01 to 128 ng/mL, with a LOD and LOQ of 0.003 and 0.01 ng/mL, respectively. In the case of IL-6, the linear working ranged from 0.5 to 1000 pg/mL, with a LOD and LOQ of 0.15 and 0.5 pg/mL, respectively. In both cases, the LOQ and the linear response range allow the direct measurement (no dilution is required) of the clinically relevant concentrations in neonatal samples. The LODs obtained for the determination of both proteins, PCT and IL-6 were found in the same order as in their individual determinations [40, 41], which indicated that the dual assay exhibited excellent sensitivities as well. The most important characteristic of the concentration linear ranges for both proteins is that they cover the required clinical ranges without prior dilution of the clinical sample. In case of PCT, although the method was linear up to 1280 ng/mL as demonstrated in previous individual aptassay, the concentration linear working interval was adjusted only up to 128 ng/mL because this interval covered the levels of this biomarker in the clinical samples.

**Table 2:**
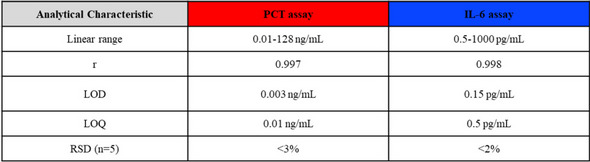
Analytical characteristics for *on-the-fly* PCT and IL-6 dual determination

Also, Fig. 4B shows how the analytical responses for the samples overlapped in both protein calibration plots, indicating that no matrix effect was observed, as expected since the sample matrix was identical to those analyzed in the previous individual assays, in which no matrix interference was observed [40, 41]. In addition, calibration recorded with the clinical samples (sample signal vs sample concentration) exhibiting a slope value of (230 ± 50) mL/ng (*r* = 0.990) for PCT and (180 ± 10 mL/pg (*r* = 0.998) for IL-6, which had not shown significant differences that obtained for their correspondence standard solutions prepared in phosphate-buffered (270 ± 40) mL/ng, *r* = 0.997; (180 ± 20) mL/pg, *r* = 0.998), for PCT and IL-6, respectively (*p* < 0.05). Therefore, no matrix effect was confirmed.

Precision was also evaluated at the minimum and maximum concentration of both proteins (0.01 and 128 ng/mL in PCT, 0.5 and 1000 pg/mL in IL-6), respectively; reaching a value of RSD_0.01_ = 0.1 and RSD_128_ = 3% (*n* = 5) in PCT, RSD_0.5_ = 2 and RSD_1000_ = 2% (*n* = 5) in IL-6. In addition, the MM batch can be reused due to the magnetic properties of the MM due to its Ni layer, which allows its withdrawal from the solution and the possibility of being used again. However, it does not make much sense, since batches with high reproducibility are obtained that contain large amounts of MM allowing a single use of them.

The simultaneous determination of PCT and IL-6 through a dual aptassay is recorded in video S1. Please note the video is exhibiting fluorescence monitoring as an analytical signal. To do this, the microscope focuses on the plane (2D) of the volume solution (3D), which is the reason why the MMs are observed in the background (optically blurred). MMs swim inside the drop (3D), and they will hardly be observed in the 2D plane, only when they navigate in the fluorescence analysis plane. The specific affinity interaction with the corresponding aptamer quenched on the MM_GO_ surface (OFF) causes its desorption and the recovery of the tag’s fluorescence (ON). The fluorescence measurement of each protein was carried out with a simple filter change, allowing the easy detection of both proteins without loss of sensitivity. Video S1 also shows the MM propulsion in the solution (3D) in the insets. Although the speed diminishes in serum samples from 80 μm/s (in buffer) to 60 μm/s, this fact does not affect the great capabilities of MM for the analysis of clinical samples. Figure S4 shows the navigation time-lapse microscopy images of MM taken at 15 min. from video S1. The dual fluorescence detection signals, related to the presence of both proteins, are recorded simultaneously at their specific emission wavelength.

### MM-based dual aptassay: clinical sample analysis

**Fig. 5:**
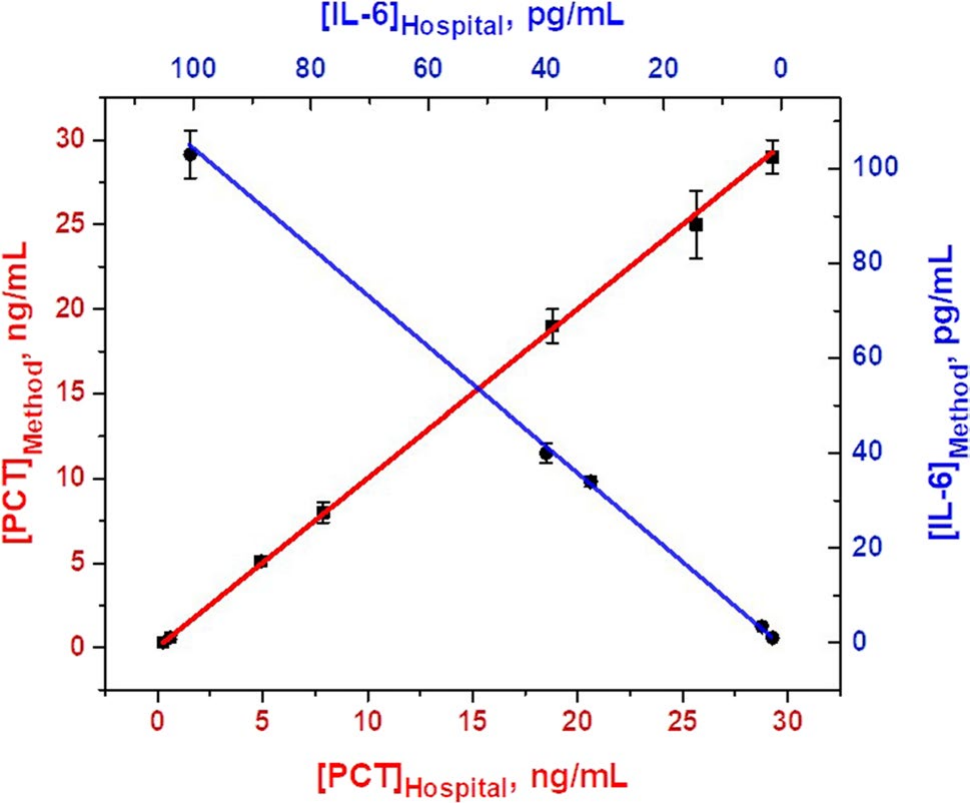
Correlation plots between the MM-based approach (y-axis) and the Hospital Method (x-axis) for PCT and IL-6 detection, *n* = 3. The conditions used are listed in Table 1

After demonstrating the excellent analytical performance of the dual aptassay, clinical samples from extremely low birth weight neonates diagnosed with sepsis at the NICU, where the volume sample available is extremely scarce and accuracy and promptness are essential, were carefully analyzed. The quantitative results obtained by the MM-based dual aptassay and the clinical reference method are listed in Table S1. Figure 5 shows the correlation plots between both sets of data, exhibiting an excellent correlation performance (*p* < 0.05) of the MM-based approach with the Hospital method for both biomarkers ((− 0.03 ± 0.02) + (1.00 ± 0.02)[PCT], *r* = 0.9992; (− 0.5 ± 0.3) + (1.05 ± 0.06)[IL6], *r* = 0.9990). Also, the results revealed an excellent reliability with low systematic error for both biomarkers and all ranges of concentration assayed (Er < 6% for PCT, and Er < 4% for IL-6) between the results obtained in MM-based dual aptassay and those reported by the Hospital laboratory (chemiluminescent immunoassay using Cobas® e411 analyzer; 30 µL of sample, 18 min for PCT and 18 min for IL-6). In control 2 for IL-6, we did not get any signal through our approach (neither in the hospital method), while for PCT we obtained a result with a good agreement compared with the clinical reference method. These results are highly relevant as accurate simultaneous determination of IL-6 and PCT levels in ultra-low blood sample volumes, in only a few minutes, is feasible with the dual aptassay in a target population of extremely low birth weight infants. More importantly, these key features could also allow the analysis of these proteins throughout the entire diagnostic window: from suspicion to confirmation of sepsis.

Related to the comparison of our reported work with others described in the literature for the determination of these biomarkers, it is important to remark that the dual PCT and IL-6 aptassay, using MM, is pioneering and a direct comparison with other technologies is not fully pertinent. Interestingly, the technological asset of this work has been to demonstrate the reliable and simultaneous determination of both PCT and IL-6 proteins in the same clinical sample, without loss of analytical performance compared to single determinations [40, 41], even slightly improved also in terms of sample volume (only 2 µL) and analysis times (15 min.).

Our MM-aptassay-based approach turned out also to be very competitive in comparison with the (few) relevant works found in the bibliography for the simultaneous determination of PCT and IL-6 using other analytical approaches. Indeed, we have obtained better sensitivities, improved analysis times, and even the use of smaller volumes of required samples [25–27]. In addition, in our approach, a set of unique real samples of neonates were analyzed, which gives exceptional value to these results and allows us to foresee a high potential of MM technology, despite still being in an adolescent stage. The proposed approach also encompasses distinctive technical attributes. Specifically, its minimal sample volume requirements and expeditious results would enable the monitoring of both sepsis biomarkers within the initial hours after the manifestation of symptoms in high-risk neonates. By obtaining minute quantities of blood, for example by heel puncture, the feasibility of even hourly assessments is facilitated. This real-time monitoring of biomarker progression may serve as a valuable tool in facilitating prompt and well-informed decisions about the initiation of antibiotic therapy.

## Conclusions

A fluorescence dual MM-based aptassay for simultaneous, fast, and accurate determination of highly relevant biomarkers such as PCT and IL-6 in clinical samples coming from neonates with gestational age less than 32 weeks and birthweight below 1000 g with suspected (late-onset) sepsis, has successfully been developed, making MM technology a promising and competitive tool in diagnosing of this relevant disease. We envision that to improve diagnostics, we should explore the best and newest health initiatives in technology and sepsis. With this in mind, we seek to initiate a true change in the paradigm to identify, analyze, and spread these new technologies.

Also, these results obtained for a truly dual assay performed in the ultra-miniaturized clinical neonatal sample environment, indicated the analytical capabilities of MM technology for multiplexing analysis, even toward the assessment of several proteins throughout the entire diagnostic window, from suspicion to confirmation of sepsis: opening new avenues in low volume-based diagnostics. This also opens the way for the development of a bedside/point-of-care tool for the diagnosis of sepsis in critically ill patients when blood sample availability is scarce and timely recognition of the problem can be lifesaving. MM technology in diagnosing (neonatal) from early to late-onset sepsis is already advancing a promising future, but we are still at the beginning of this adventure.

### Supplementary Information

Below is the link to the electronic supplementary material.

Supplementary file1 (DOCX 370 KB)

Supplementary file2 (MP4 5424 KB)
