# Supplementary material for: Micromotor-based dual aptassay for early cost-effective diagnosis of neonatal sepsis
Source: Mikrochim Acta. 2024 Jan 19;191(2):106. doi: 10.1007/s00604-023-06134-x (PMC10798920; doi:10.1007/s00604-023-06134-x)
Supplement: Supplementary file 1 — Supplementary file1 (DOCX 370 KB) [file 604_2023_6134_MOESM1_ESM.docx]

**Supplementary Information**

**Micromotors-based dual aptassay for early cost-effective diagnosis of neonatal sepsis**

José M. Gordón Pidal ^a^, Luis Arruza ^b^, María Moreno-Guzmán ^c^, Miguel Ángel López ^a,d,*^, Alberto Escarpa ^a,d,*^.

1. Department of Analytical Chemistry, Physical Chemistry and Chemical Engineering, University of Alcalá, Ctra. Madrid-Barcelona, Km. 33.600, Alcalá de Henares, 28871, Madrid, Spain.
2. Department of Neonatology, Instituto del Niño y del Adolescente, Hospital Clínico San Carlos-IdISSC, 28040 Madrid, Spain.
3. Department of Chemistry in Pharmaceutical Sciences, Analytical Chemistry, Faculty of Pharmacy, Complutense University of Madrid, Plaza Ramón y Cajal, s/n, 28040 Madrid, Spain.
4. Chemical Research Institute “Andrés M. Del Rio”, University of Alcalá, Madrid, Spain.

**A**

**B**


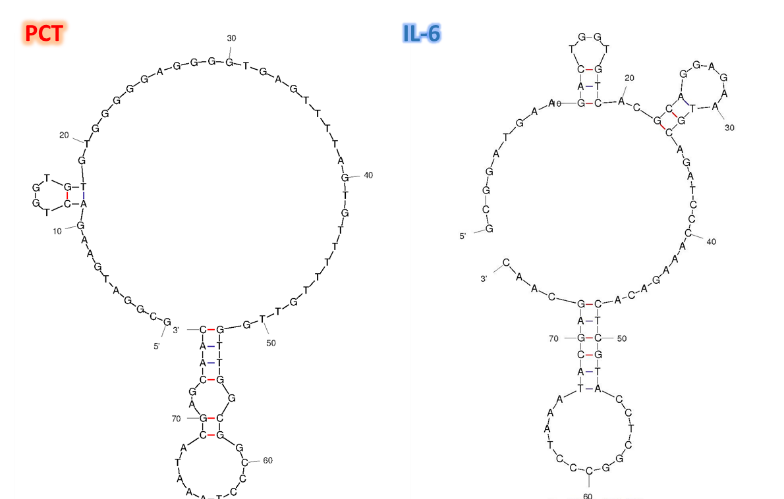


**Figure S1**. Proposed structure for PCT (A) and IL-6 (B).


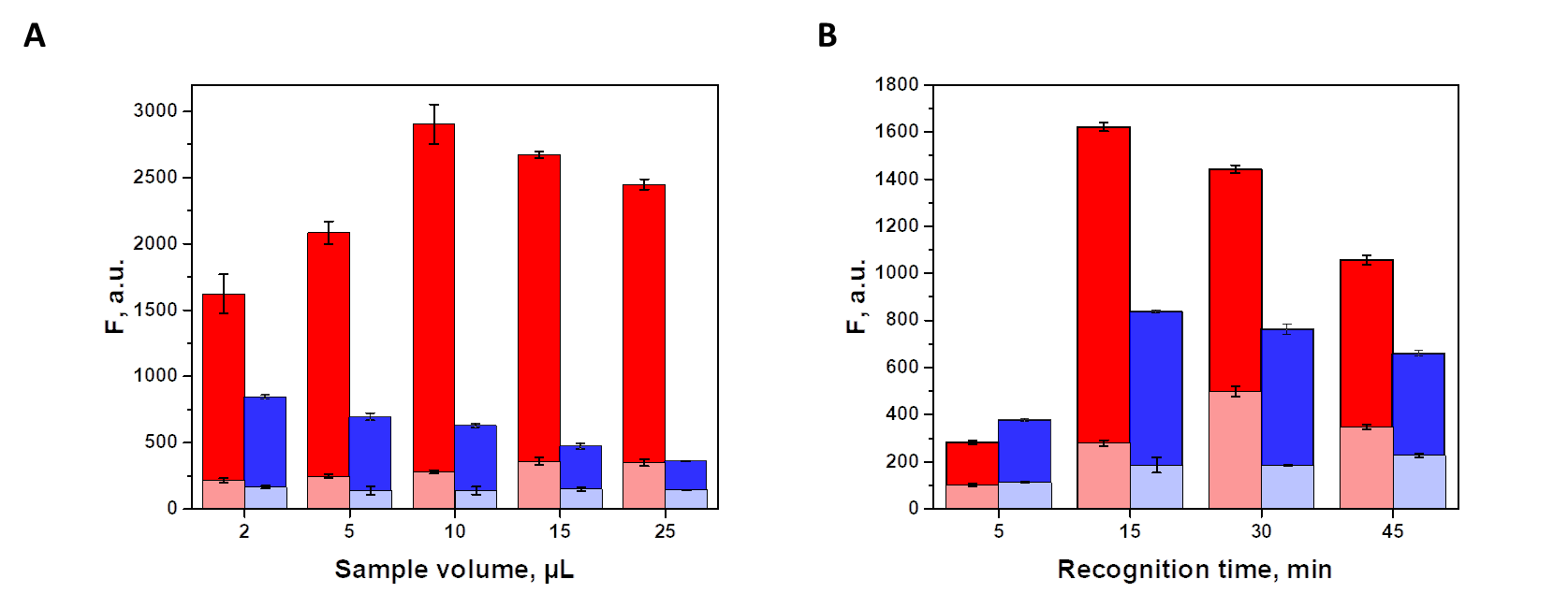


**Figure S2.** Sample volume (A) (2, 5, 10, 15, 25 µL), recognition time (B) (5, 15, 30, 45 min), for PCT and IL-6. Conditions (see Table 1). Red, and blue with light color: Controls without protein; Red and blue with dark color: *on-the-fly* aptassay strategy with an excess of PCT and IL-6 respectively, n=3.


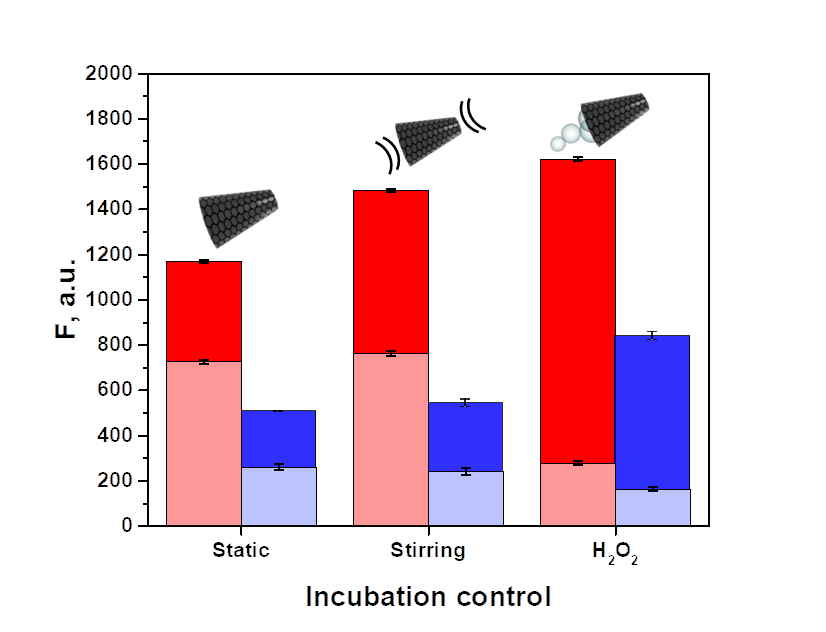


**Figure S3.** Incubation controls for PCT and IL-6 (static, stirring, bubble). Red, and blue with light color: Controls without protein; Red and blue with dark color: *on-the-fly* dual aptassay with an excess of PCT and IL-6 respectively, n=3. (Conditions, see Table 1).


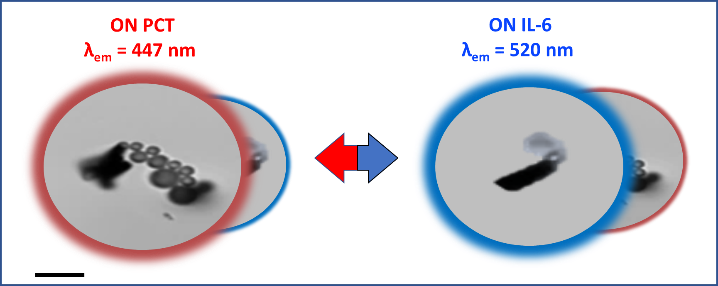


**Figure S4.** Navigation time-lapse microscopy images of micromotors, taken at 15 min. of simultaneous dual fluorescence detection of PCT and IL-6 (taken from video S1). Left: PCT detection at 447 nm (behind is IL-6 detection which is not recorded at that wavelength). Right: IL-6 detection at 520 nm (behind it is PCT detection that does not register at that wavelength). Scale 10 µm.

**Table S1.** Analysis of clinical samples from neonates with sepsis suspicion

Values are expressed as Mean Standard deviation (n=5). ND-Not Determined.
